# Optic Atrophy 1: The Conductor of Cellular Harmony and Age-Related Pathologies

**DOI:** 10.14336/AD.2025.0017

**Published:** 2026-03-19

**Authors:** Ye Xu, Jingwen Zhu, Qixiang Shao, Hui Wang

**Affiliations:** ^1^Jiangsu Key Laboratory of Medical Science and Laboratory Medicine, School of Medicine, Jiangsu University, Zhenjiang, Jiangsu, China.; ^2^Institute of Medical Genetics and Reproductive Immunity, Jiangsu Province Engineering Research Center of Precise Prevention in the Digestive and Reproductive System Cancers, Jiangsu College of Nursing, Huai’an, Jiangsu, China.

**Keywords:** OPA1, Mitochondria, Mitochondrial dynamics, Age-related diseases

## Abstract

As the population aging, the prevalence of age-related diseases is also rising. Mitochondrial malfunction is one of the hallmarks of aging, and optic atrophy type 1 (OPA1), a protein found in the inner membrane (IM) of mitochondrial, is essential to this process. OPA1 regulates the fusion of IM and cristae structure, hence maintaining cellular energy metabolism and function. Its abnormalities may impair the multiple functions of tissues and are also closely related to various diseases. OPA1 is highly expressed in metabolically active organs, such as the brain, skeletal muscle, and heart, ensuring the normal metabolism and function of these organs. This review summarizes the physiological functions of OPA1 in these organs, along with the effect of aberrant OPA1 expression on aging related disorders. By deeply studying the mechanisms of OPA1’s function in these diseases, we might achieve a more profound comprehension of the pathological processes of age-related diseases and explore potential therapeutic strategies.

## Introduction

**Figure 1.**
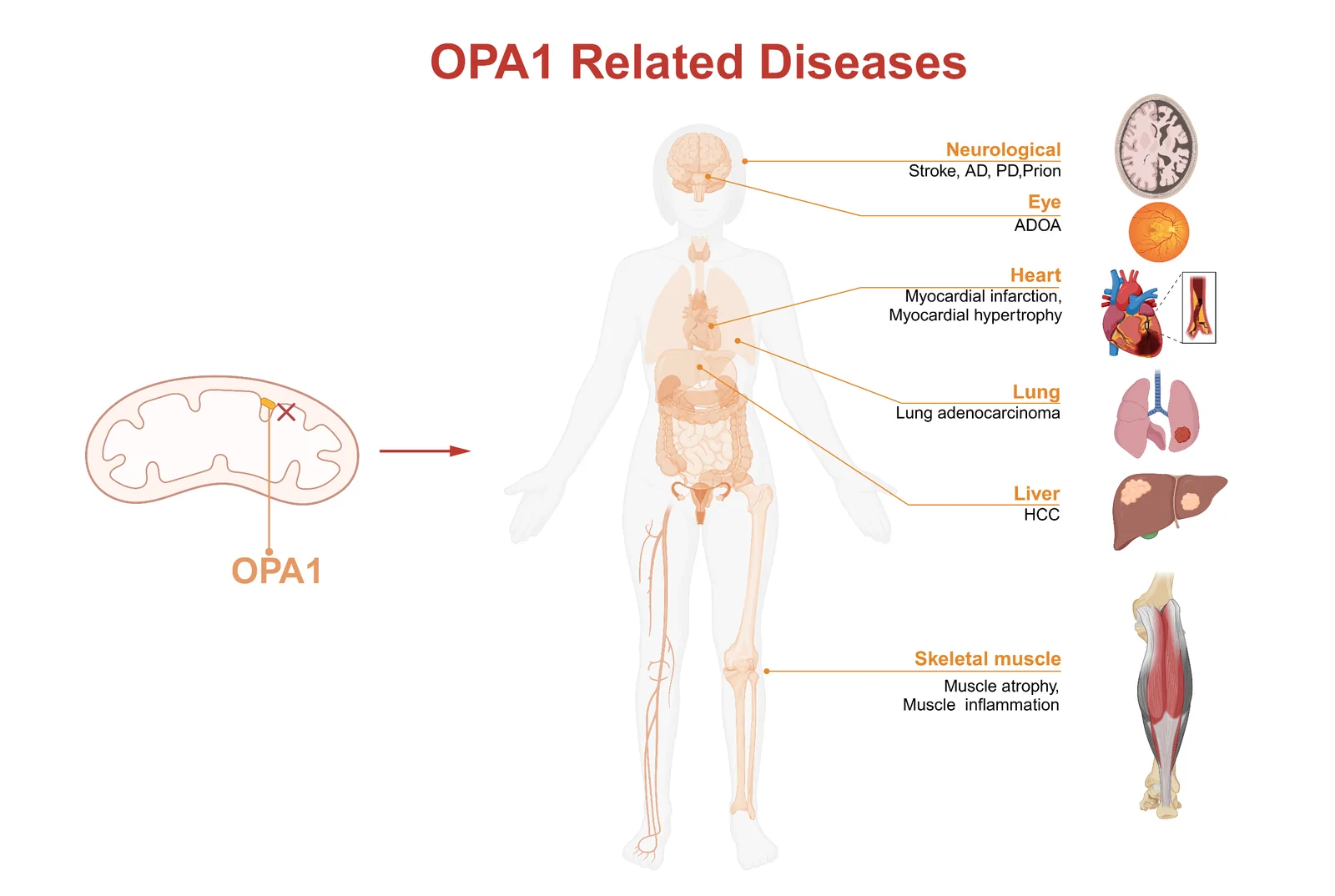
**OPA1 related diseases.** OPA1 is widely expressed across various organs, its dysfunction has been implicated in the pathogenesis of numerous diseases. AD, Alzheimer’s disease. PD, Parkinson's disease. ADOA, autosomal dominant optic atrophy. HCC, hepatocellular carcinoma.

Mitochondria originated from bacteria and exist symbiotically within eukaryotic cells [1]. As energy factories in eukaryotic cells, the number and function of mitochondria fluctuate greatly across different organisms and tissue types to accommodate diverse energy demands. Except for mature red blood cells, all cells in mammals contain mitochondria, with their numbers ranging from tens to thousands [2]. Mitochondria are particularly important in high-energy-demanding tissues, such as the heart and brain, and their dysfunction is related to numerous human diseases. To adapt to changes in cellular metabolism and physiology, mitochondrial activity needs to be strictly monitored and optimized. Age-related changes in mitochondrial dynamics may lead to severe consequences, such as mitochondrial dysfunction, characterized by impaired mitochondrial fission, enlarged mitochondria, and decreased autophagy efficiency [3]. As the global aging process accelerates, the connection between mitochondrial dynamics and aging is attracting heightened scrutiny. Maintaining the structural integrity of mitochondria depends upon the activity of OPA1 protein and its regulatory mechanisms. OPA1 plays a crucial role in mitochondrial energy metabolism, where it is involved in mitochondrial dynamics, including fusion, fission, and maintenance of cristae structure. Alterations in OPA1 are intimately related to the aging process. The decrease of OPA1 protein during aging is considered a contributing factor to age-related functional impairment, including vision loss, muscle weakness, and memory impairment [4]. The absence of OPA1 protein can also affect the characteristics and self-renewal capacity of stem cells, leading to a gradual loss of adult stem cells with age [5]. Furthermore, alterations in OPA1 are associated with inflammatory responses. Abnormalities in OPA1 are linked to various age-related diseases, including neurodegenerative disorders and cancer (Fig. 1). This review will provide a detailed overview of the molecular processes involving OPA1 in health, aging, and regeneration, as well as an exploration of the current understanding of the therapeutic applications.

### 1. Mitochondria and its fusion protein OPA1

#### 1.1. Mitochondrial structure and its role in cell health

**Figure 2.**
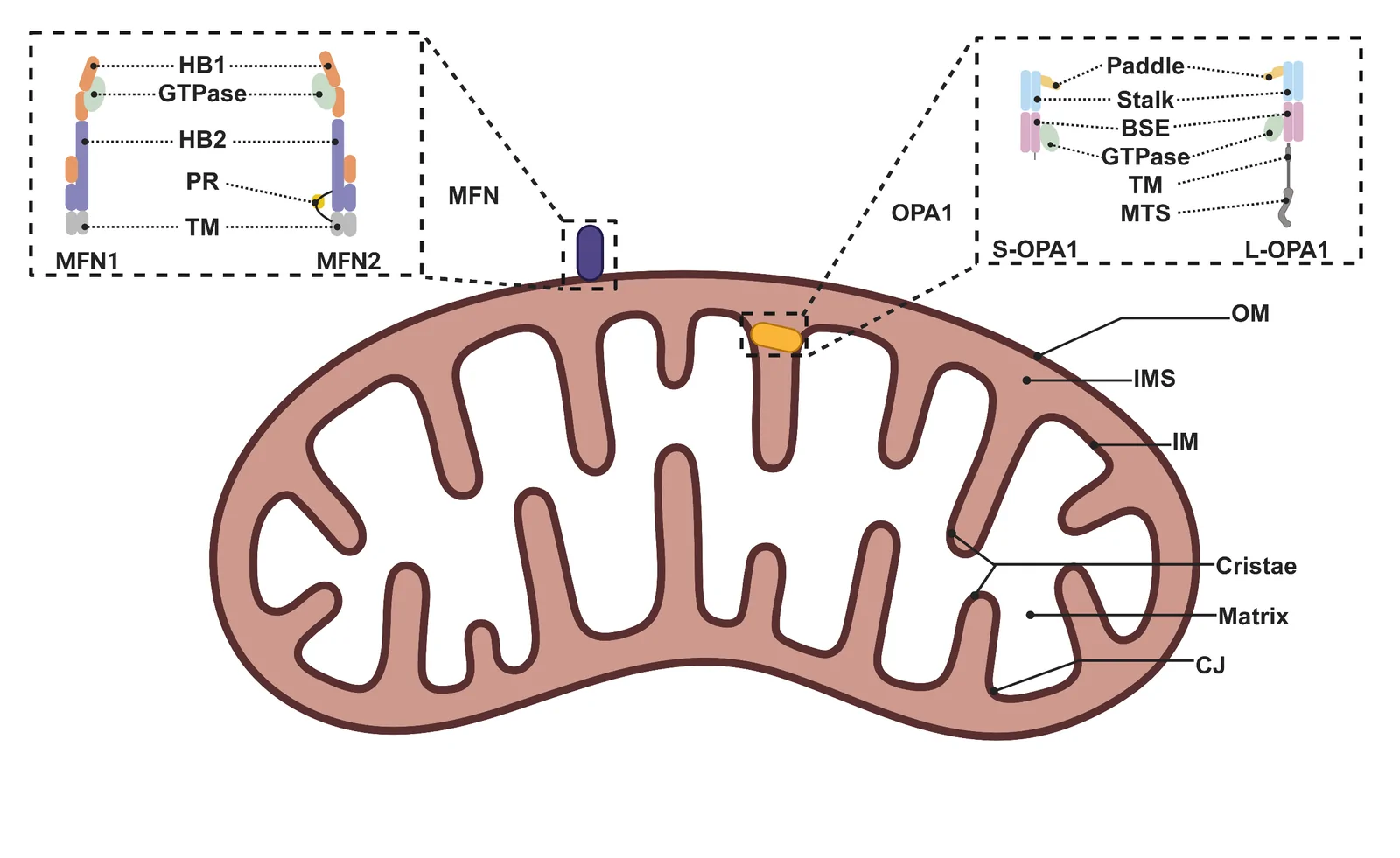
**Mitochondrial fusion proteins.** MFN1/2 are fusion proteins located on the outer membrane (OM), while OPA1 is a fusion protein associated with the inner membrane (IM). The magnified regions of OPA1 and MFN1/2 highlight their distinct structures, with different colors representing various domains. This includes helix bundle 1 (HB1), helix bundle 2 (HB2), proline-rich domain (PR), transmembrane domain (TM), bundle signaling element (BSE), and mitochondrial targeting signal (MTS). The inner membrane space (IMS) and the cristae junction (CJ) are also indicated.

Mitochondria are double-membrane structures, including outer membrane (OM) and IM. The region between these two membranes is called the intermembrane space (IMS), which acts as a separator between OM and IM that encases the mitochondrial matrix (Figure 2). The double-membrane structure of the mitochondria is critical to their function. The OM is porous, allowing small molecules and ions to flow freely, such as the voltage-dependent anion channel (VDAC), while larger molecules require specialized translocases to import [6]. Nevertheless, the IM is a stringent permeability barrier that creates an electrochemical gradient, driving oxidative phosphorylation (OXPHOS) to produce ATP. The respiratory complexes on the IM generate a transmembrane potential *via* electron transport and/or proton pumping, which fuel ATP synthesis [7, 8]. Mitochondrial cristae are specialized structures of the IM that increase the surface area of membrane to enhance the process of OXPHOS, which is a key stage in mitochondrial energy production [9]. The integrity of the cristae directly affects the efficiency of OXPHOS, as it is home to several major molecular complexes of the electron transport chain (ETC) and OXPHOS [10–12]. Therefore, when the structure or number of cristae changes, respiratory complexes become unstable and start to break down. This might have an impact on energy metabolism and potentially trigger apoptosis.

#### 1.2. Mitochondrial dynamics and its regulation

Mitochondrial dynamics, encompassing fusion, fission, transport, and turnover, or called selective degradation, play a vital role in preserving the health of the mitochondrial network. Mitochondrial fission is mainly regulated by dynamin-related protein 1 (DRP1) [13]. DRP1, which is recruited from the cytosol to the OM, assembles into ring-like or helix-like structures that lead to mitochondrial fraction [14]. This process requires the assistance of proteins such as MFF, MiD49, and MiD51, as well as the involvement of the endoplasmic reticulum (ER) [15–17]. Two main proteins in mammals regulate mitochondrial fusion: Mitofusins (MFN1 and MFN2) anchored to the OM and OPA1 located in the IM (Figure 2). Mitofusins facilitate the fusion of the outer membranes, whereas OPA1 manages the integration of the IM [18–20]. These two membrane fusion events occur close together in time but are spatially and temporally separated [21]. The ultimate result of mitochondrial membrane fusion is matrix contents mixture [22]. OPA1 is an indispensable protein in mitochondrial dynamics, with functions that include mitochondrial morphology, fission and fusion, cristae structure regulation, and apoptosis [23].

#### 1.3 The role of OPA1 and its isoforms in mitochondrial function and cleavage

**Figure 3.**
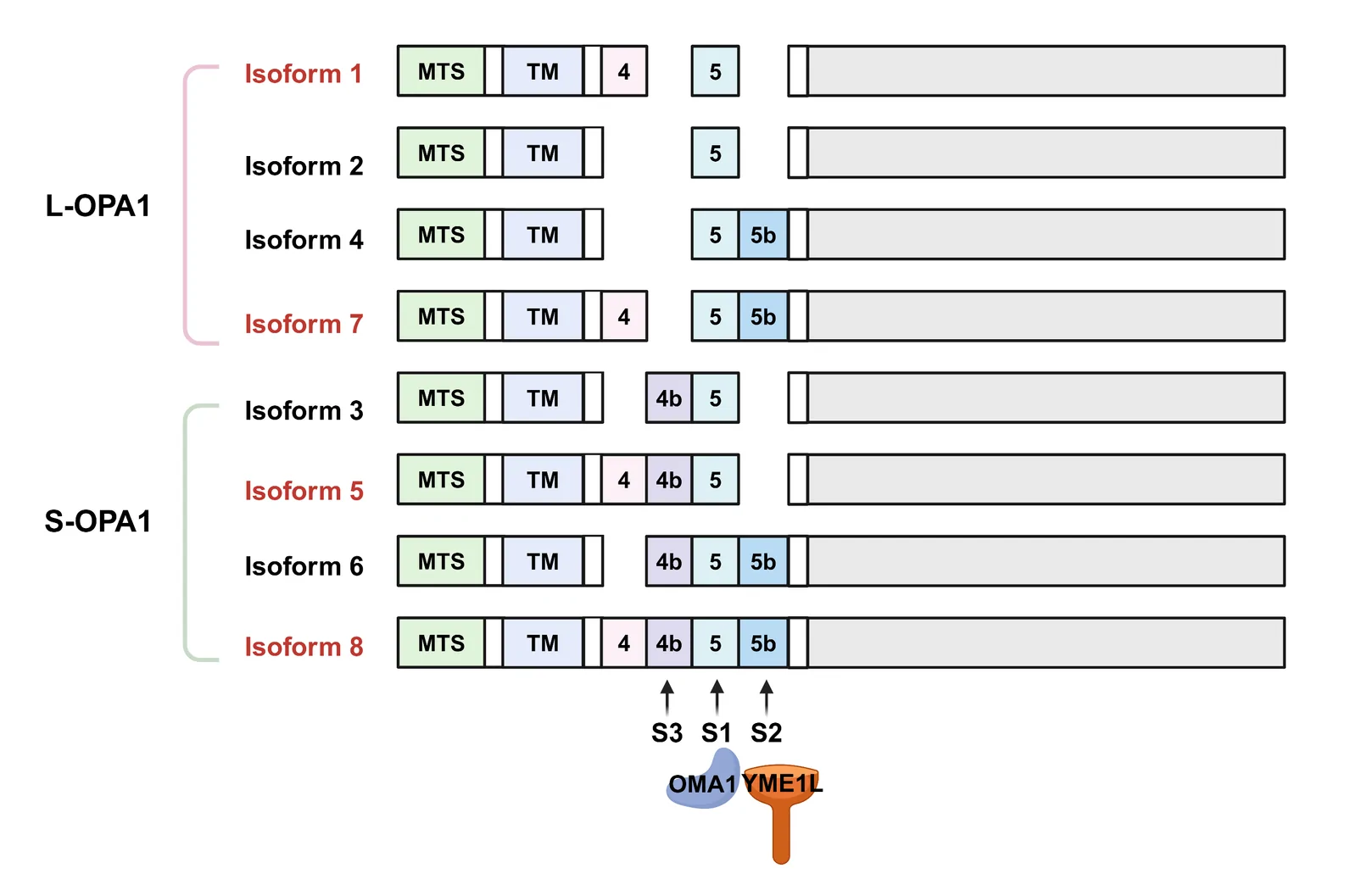
**OPA1 cleavage sites and isoforms.** Human OPA1 has eight isoforms, while mouse has four isoforms (labeled in red). The splicing site is localized to different exons, and OPA1 can be divided into different isoforms based on different combinations of exons 4, 4b, 5, 5b. Isoforms 1, 2, 4 and 7 produce L-OPA1, which can be cleaved into both long and short isoforms, while isoforms 3, 5, 6 and 8, which carry Exon 4b, produce S-OPA1 only. The OMA1 protease cleaves OPA1 at the S1 site, while YME1L cleaves at the S2 site. MTS, mitochondrial targeting signal. TM, transmembrane domain. S1, cleavage site 1. S2, cleavage site 2. S3, cleavage site 3.

The OPA1 gene, situated on the 3q28 chromosome band, comprises 31 exons and spans a total length of 100 kb [24, 25]. Through alternative splicing of exons 4, 4b, and 5b, the human OPA1 gene produces eight different isoforms. Each isoform expresses itself differently in various tissues (Figure 3). Olichon et al. found that exon 4 plays a vital role in modulating mitochondrial morphology and is a key participant in the process of mitochondrial fusion. Silencing OPA1 variants that contain this exon results in the fragmentation of the mitochondrial network, with mitochondria becoming spherical and significant dissipation of mitochondrial membrane potential (MMP). Exons 4b and 5b are related to apoptosis, and silencing OPA1 variants that contain either of these exons results in chromatin condensation and fragmentation [26]. Exon 4b is also linked to changes in mitochondrial DNA (mtDNA). The suppression of OPA1 variants containing Exon 4b results in a reduction of mtDNA and a substantial perturbation in the distribution of mtDNA within the mitochondrial network [27]. According to the presence of exon 4b, the eight human isoforms can be divided into isoforms 1, 2, 4, 7, which produce the long form isoform of OPA1 (L-OPA1) and the short form isoform of OPA1 (S-OPA1), and isoforms 3, 5, 6, 8 which produce only S-OPA1 [28]. In mouse, there are only four isoforms, isoforms 1, 5, 7, 8 [29]. Exon 5 is a constant component across all isoforms and contains the sequence for the S1 cleavage site. This site is recognized by the metalloprotease OMA1, which locates in the IM [30]. Under mitochondrial stress conditions, such as loss of MMP or ATP, the OMA1 protease becomes activated. This activation cleaves OPA1, leading to its inactivation. This rapid and complete cleavage affects all OPA1 isoforms that yeast mitochondrial escape1-like ATPase (YME1L) has not yet acted upon, thereby impacting metabolic function and mitochondrial morphology. This process is ATP-independent [30–33]. Exon 5b is present in isoforms 4, 6, 7, 8, encoding the S2 cleavage site. This site is recognized by the mitochondrial matrix protease YME1L [34]. YME1L is localized to the mitochondria, with its catalytic domain facing the mitochondrial intermembrane space. It manages the processing and stability of OPA1 through its proteolytic activity [35]. The processing of OPA1 by mitochondrial metalloproteases is regulated by the mitochondrial IM potential (ΔΨm). When ΔΨm is lost, such as treating with carbonyl cyanide m-chlorophenyl hydrazone (CCCP), it triggers a swift conversion of L-OPA1 to S-OPA1. This process may be mediated by YME1L [36]. Wang et al. identified a new cleavage site, S3, in the C-terminal leucine string of OPA1 exon 4b. This site is located just upstream of the known S1 site, and relies on the YME1L protease [37]. Additionally, the splicing of OPA1 is regulated by prohibitin (PHB) and peroxisome proliferator-activated receptor gamma-like protein kinase 2 (PARL). The absence of PHB activates OMA1, leading to the degradation of L-OPA1 [38, 39]. PHB stabilizes cardiolipin (CL) within the mitochondria, thereby regulating OMA1 and subsequently affecting its cleavage activity on OPA1 [40]. PARL, as a mitochondrial protease, participates in the regulation of the balance between the long and short isoform of OPA1 through its proteolytic activity, and then affecting mitochondrial morphology and apoptosis [41]. Some studies have indicated that under specific conditions, when OMA1 is activated and the expression of PARL is reduced, OMA1 may partially take over the role of PARL [42]. PARL, along with stomatin-like protein 2 (SLP2) and YME1L, may constitute a large protease complex termed the SPY complex. YME1L is also referred to as the i-AAA protease. SLP2 can limit the processing of OPA1 by OMA1 [43]. The protein bands of OPA1 typically consist of five bands, with different splice variants showing distinct bands. Bands a and b are a mixture of L-OPA1, bands c and d represent the products after S1 and S2 cleavage, and band e is a form of S-OPA1 [37, 44].

#### 1.4. The function of OPA1 in mitochondrial dynamics

Mitochondria maintain the dynamic equilibrium of their network through fission and fusion, with OPA1 serving as a crucial regulator in this process. L-OPA1 is central to mitochondrial fusion. It is anchored to the IM and promotes the approach and fusion of two mitochondrial membranes through its GTPase activity. Although S-OPA1 does not directly fuse membranes, it may assists L-OPA1 in the fusion process [45]. Although OPA1 is primarily associated with mitochondrial fusion, its proteolytic product S-OPA1 may be involved in the fission process. Under certain conditions, the accumulation of S-OPA1 is associated with mitochondrial fragmentation [44, 46]. Recent researches indicate that S-OPA1 contributes to the fusion of the IM, possibly related to specific combinations of OPA1 isoforms [28], or the ratio of L-OPA1 and S-OPA1 [37, 47]. The fusion function of OPA1 is also related to CL, a phospholipid found in mitochondrial. Studies have discovered that the protein OPA1 and CL act in synergy in the fusion of the heterogeneous IM. Experiments suggest that L-OPA1 on one membrane surface and CL on the opposite surface are adequate for promoting mitochondrial fusion [48]. CL can specifically stimulate the GTPase activity of OPA1, through specific binding sites, and is involved in fusion [49]. Recent study has revealed that the paddle domain of OPA1 plays a crucial role in membrane binding and membrane remodeling. OPA1 inserts itself into the CL-rich membrane through its lipid-binding paddle domain, where a conserved loop (L1P) penetrates deeply into the membrane bilayer, thereby stabilizing interactions with cardiolipin. OPA1 promotes the helical assembly of a flexible OPA1 lattice on the membrane through paddle domain dimerization, which drives mitochondrial fusion [50]. Abnormal OPA1 function may alter mitochondrial morphology, causing excessive fusion, which results in a reticular structure or excessive fission, leading to fragmentation. When OPA1 function is impaired, the ability of the IM to fuse decreases, leading to mitochondrial fragmentation and reduced efficiency of OXPHOS [51]. In addition, exon 4b of OPA1 mediates a novel mitochondrial function repair mechanism that is independent of mitochondrial fusion. When OPA1 contains exon 4b, it can specifically bind to a special part of mtDNA called the D-loop. This helps repair the ETC by promoting the transcription and translation processes of mtDNA, which in turn restores mitochondrial respiratory function [52]. Therefore, understanding the molecular mechanisms that control mitochondrial morphology, and function plays a vital role in the progress of therapeutic strategies. The dynamic equilibrium of the mitochondrial network is the foundation of cellular health and adaptation to environmental changes, and OPA1 is a key factor in this process.

#### 1.5. The impact of OPA1 in mitochondrial cristae morphology and function

The lack of OPA1 affects the structure of the cristae and subsequently impacts energy metabolism [53]. OPA1 maintains not only the structure of the cristae, but also the morphology of mitochondria. In addition, this function can be separated from mitochondrial fusion [54, 55]. OPA1 responds to changes in the levels of fuel substrates through the solute carrier family 25A (SLC25A), and then regulating the structure of the cristae and the assembly of ATP synthase, which affects mitochondrial function [54]. Cristae have a variety of morphologies, such as flat plate-like or tubular structures [56, 57]. Numerous variables, including the action of the OPA1 protein, govern these morphologies. An analysis of the cristae’s structure reveals that OPA1 promotes the tubular cristae while inhibiting plate-like cristae. Research has revealed that the proportion of tubular structures in OPA1 knockdown cells’ mitochondria is considerably lower [58]. Moreover, OPA1 overexpression can ameliorate the phenotype of mitochondrial diseases by improving the cristae shape in mice [59]. According to the research by Fry et al., OPA1 controls the width of the cristae junctions (CJs), the sites at which the cristae membrane contacts the inner boundary membrane (IBM) [60]. During apoptosis, the OPA1 complex breakdown allows the cytochrome c (Cyt c) getting away from the inner space of the cristae, which is typically followed by morphological changes in the CJs [55, 61]. However, different studies hold different opinions on the specific changes in the CJs during apoptosis. According to Frezza et al., OPA1 controls the progress of Cyt c releases in period of apoptosis by regulating the morphology of the cristae and preventing the overly wide CJs. This function is independent in mitochondrial fusion [55]. In contrast, The results of Yamaguchi et al.’s show that OPA1 is degraded and CJs become narrow under the treatment of BH3-interaction domain death agonist (BID) [62]. These findings underline the pivotal function of OPA1 in maintaining cellular health and adaptability and regulating the structure and function of mitochondrial cristae.

### 2. The role of OPA1 in muscle vitality, aging, and regeneration

Skeletal muscle stem cells (MSCs), alternatively referred to as satellite cells, remain mostly quiescent throughout life. It can be easily activated by injury or stress signals, then participate in myogenic programs, which is a key step of skeletal muscle regeneration [63]. Stem cell exhaustion is one of the hallmarks of aging [64]. In mature mouse muscle, satellite cells are usually in a mitotically quiescent state [65]. The quiescent stage of stem cells is a critical state in the cell cycle. This stage is in a reversible non-proliferative phase and maintains active metabolic activity, allowing them to sustain tissue homeostasis and regenerative capacity over the long term [66]. Satellite cells possess the ability for self-renewal, can divide to produce more satellite cells, while maintaining their stem cell characteristics, playing a key role in muscle injury and regeneration [67]. The proliferation and differentiation of satellite cells are crucial for muscle repair after injury, they aid in the recovery of muscle, and possess the ability to regenerate muscle [68]. MSCs shift towards glycolysis and glutaminolysis when changing from a quiescent phase to a proliferative phase. Mitochondria influence the self-renewal, differentiation, and tissue regenerative ability of stem cells by regulating energy metabolism and histone acetylation levels [69]. Changes in mitochondrial function and metabolic state affect the self-renewal and differentiation capacity of skeletal MSCs, thereby affecting muscle development and regeneration after injury [70]. The number of satellite cells decreases with age, although they still maintain the ability to expand and differentiate through the myogenic program [71]. Satellite cells in aged and post-mortem tissues exhibit a decrease in mitochondrial function and a diminished capacity in energy production. Proliferating satellite cells during the muscle regeneration consume more oxygen and have a higher ATP content than quiescent cells [72]. Autophagy activity is constitutive, while it is impaired in aged cells. Restoring autophagy activity in aged satellite cells can prevent cellular senescence and restore their proliferative and regenerative capacities. Hence, autophagy is critical in maintaining the quiescent state of MSCs and preventing senescence [73]. The regenerative capacity of satellite cells is largely dependent on mitochondrial dynamics. As age or genetic damage progresses, mitochondrial fission in these cells is reduced, resulting in the impairment of the mitochondrial ETC, reduced OXPHOS metabolism and mitochondrial autophagy efficiency, increased oxidative stress, and muscle regeneration failure. Restoring regenerative in damaged or aged satellite cells involves re-establishing mitochondrial dynamics, OXPHOS, or mitophagy [74].

#### 2.1. OPA1 as the guardian of skeletal muscle regeneration

**Figure 4.**
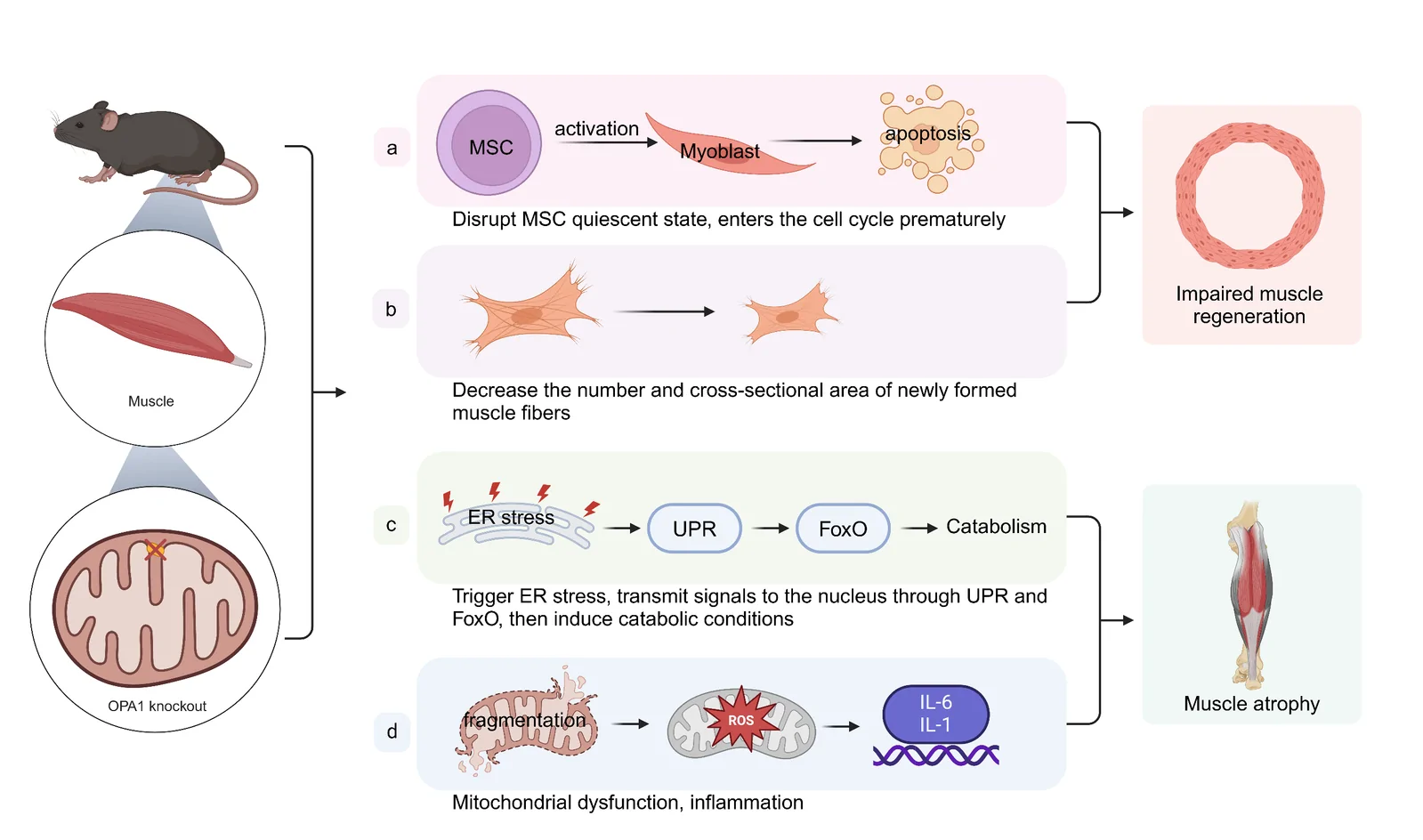
**OPA1 deficiency leads to reduced muscle regeneration and muscle atrophy.** The absence of OPA1 leads to **(a)** a decrease in the maintenance of MSCs, which advances the cell cycle and subsequently impairs the muscle regeneration; **(b)** a reduction in both the number and cross-sectional area of newly formed muscle fibers, thereby impairing muscle regeneration; **(c)** ER stress, which activates UPR and FoxO, inducing a catabolism program that results in muscle atrophy; **(d)** mitochondrial fragmentation may increase the production of ROS, which then activate inflammatory cytokines and contribute to muscle atrophy.

OPA1 and mitochondrial dynamics, play a regulatory role in adult MSCs quiescent state [75]. The absence of OPA1 leads to mitochondrial dysfunction, which disrupts the resting state of MSCs, making them highly sensitive and prone to activation, accelerating the cell cycle process, and impairing stem cells maintenance (Fig. 4a). Case reports demonstrate that when individuals have homozygous OPA1 mutations, their muscle tissue samples reveal severe mtDNA loss and a comprehensive decrease in mitochondrial respiratory chain complex activity. This leads to neuromuscular weakness and death [76]. The absence of OPA1 significantly reduces the capacity for muscle regeneration, resulting in a reduction of the quantity and cross-sectional area of newly formed muscle fibers, severe impairment of myoblast differentiation, and consequently affecting muscle mass and function (Fig. 4b). OPA1 promotes myocytes differentiation by facilitating the redistribution of mitochondrial structure and cristae architecture, and regulating the expression of SR-Related CTD Associated Factor 1 (SCAF1). SCAF1 is a super-complex assembly factor associated with metabolic reprogramming. OPA1 is particularly important in muscle regeneration and differentiation, making it a viable target for the treatment of muscle diseases [77]. Macrophages are important to muscle regeneration. They establish a microenvironment that is favorable for the activation, proliferation, and differentiation of muscle satellite cells, by switching between pro-inflammatory and anti-inflammatory states. OPA1 regulates macrophages’ metabolic activity and function. In muscle regeneration models, knocking out the OPA1 gene impaired activation of the nuclear factor kappa-B (NF-κB) signaling pathway. This promotes the M1 polarization of macrophages, which impacts their response to inflammation and tissue repair, leading to excessive collagen deposition and impaired muscle regeneration [78].

#### 2.2. The impact of OPA1 downregulation on muscle aging

The absence or dysfunction of OPA1 is associated with age-related sarcopenia [79]. In mouse models, muscle-specific OPA1 gene knockout leads to impaired growth and eventual death in newborn mice. In adult mice, the acute knockout of OPA1 leads to muscle atrophy, decreased strength, and multi-organ functional decline, inducing a premature aging phenotype and even premature death. The absence of OPA1 triggers ER stress (ERS), transmitting signals to the nucleus through the unfolded protein response (UPR) and forkhead box protein class O (FoxO), and then inducing catabolic conditions (Fig 4c). Inhibit fibroblast growth factor 21 (FGF21) can ameliorate the aging phenotype in OPA1 knockout mice, but its effect on improving muscle mass is limited [80]. Muscle-specific knockout of OPA1 and DRP1 is embryonically lethal, hence, a tamoxifen-induced double knockout mouse model is used. When OPA1 and DRP1 are both acutely knockout, it leads to the accumulation of dysfunctional mitochondria, along with the suppression of autophagy and mitophagy processes. This, in turn, causes muscle atrophy and weakness. However, when OPA1 is depletion, the concurrent inhibition of the mitochondrial fission protein DRP1 can alleviate the induction of FGF21, oxidative stress, denervation, and inflammation, thereby saving the OPA1 knockout mice from lethality (Fig 4d). Although muscle weakness remains, the inhibition of DRP1 helps improve muscle mass and strength [81]. Consequently, the concurrent suppression of fusion and fission mechanisms alleviates the harmful consequences of unregulated mitochondrial fusion, at the same time, hinders the release of factors that promote senescence.

Muscle inflammation is an early event in the process of OPA1 deficiency, occurring before macrophage infiltration. The suppression of OPA1 in muscle cells results in a decrease in the quantity of mtDNA copies, mtDNA stress, and respiratory chain dysfunction, activation of toll-like receptor 9 (TLR9) and NF-κB, and inflammation [82]. In humans, serum levels of FGF21 increase with age. Muscle inflammation induced by OPA1 deficiency leads to a systemic inflammatory response and increased expression of FGF21, which may be a key factor in accelerating aging and metabolic changes [80].

OPA1 expression levels decline with age in the elderly population, particularly in individuals who are sedentary and at risk of sarcopenia. Regular exercise can prevent this decline in OPA1 [80]. In old rats trained in cold water swimming exhibit significantly increases in OPA1 mRNA and protein expression. Rats trained in cold water (5℃) show higher levels of OPA1 expression compared to those trained in thermoneutral water (36℃). Furthermore, female rats exhibit a more pronounced increase in OPA1 expression in response to cold water swimming training than male rats. This enhances the energy metabolism of the muscles in old rats, as evidenced by the elevated levels of muscle metabolic substrates, along with improved mitochondrial biogenesis and dynamics. These findings imply that physical activity may influence mitochondrial function and muscle health by modulating OPA1 [83].

### 3. The relationship between mitochondrial dynamics and cardiovascular diseases

The heart, being a high-energy-demanding organ, necessitates a substantial amount of ATP to preserve its regular functioning. Heart health is obviously dependent on mitochondria, the main powerhouses within the body responsible for generating energy. As we age, mitochondria get smaller, the number and morphology of cristae also change. In myocardial fibroblasts with mitochondrial contact site and cristae-organizing system (MICOS) complex gene knockout, a reduction in mitochondrial length and volume, and decreased OXPHOS function are observed [84]. Mitochondrial dynamics, which refer to the morphological changes and network balance of mitochondria, are vital for heart health [85]. Disruption of mitochondrial dynamics can exacerbate mitochondrial dysfunction, impair cardiomyocytes survival, additionally, cause ischemia/reperfusion injury (IRI), cardiomyopathy, and heart failure. Mitochondrial dynamics exhibit an intimate association with reactive oxygen species (ROS) generation and homeostasis. Notably, excessive ROS accumulation has been shown to induce endothelial dysfunction through oxidative stress-mediated pathways, a pathophysiological mechanism recognized as a pivotal driver in the pathogenesis of various cardiovascular disorders, including atherosclerosis, hypertension, and pulmonary arterial hypertension [86]. The deletion of the MFN1 gene in cardiomyocytes results in mitochondrial fragmentation, yet it is associated with preserved left ventricular function, and there are no significant differences in cardiac structure and cardiac output. These fragmented mitochondria show increased resistance to ROS-induced dysfunction and cell death. This suggests that mitochondrial fragmentation, driven by the absence of MFN1, is not sufficient in itself to induce cardiomyocyte dysfunction or mitochondrial impairment [87]. However, MFN2 deletion in cardiac cells has different outcomes. It causes abnormal mitochondrial morphology in cardiomyocytes, with pleomorphism and increased size, exhibiting a degree of myocardial hypertrophy and a slight decrease in heart function. On the other hand, adult cardiomyocytes lacking MFN2 are protected against various stimuli that induce cell death, and hearts with MFN2 gene knockout show better recovery after local ischemia and reperfusion injury [88]. MFN2 also mediates the fusion of autophagosomes with lysosomes in the heart, which is important for autophagy in the heart [89]. Compared with single fusion-defective MFN1/MFN2 or single fission-defective DRP1 gene knockout, mice with cardiac triple knockout of MFN1/MFN2/DRP1 survive longer and exhibit a distinct type of pathological cardiac hypertrophy (**Error! Reference source not found.** 1). But eventually, the simultaneous elimination of fission and fusion triggers a substantial and progressive accumulation of mitochondria, seriously distorting the sarcomeric structure of cardiomyocytes. Although the triple knockout mice show better short-term survival, in the long run, this enforced mitochondrial inactivity exacts a toll on mitochondrial quantity and accelerated the aging progress of the mitochondrial [90].

**Table 1.** Effects of different gene treatments on mitochondrial and cardiac phenotype.

Treatment		Results	
Gene	Technique	Mitochondrial morphology	Heart phenotype
**MFN1**	Knockout	Mitochondrial fragmentation	Normal cardiac function [87]
**MFN2**	Knockout	Pleomorphism, enlarged mitochondria	Myocardial hypertrophy, slight decrease in heart function [88]
**MFN1, MFN2, DRP1**	Trible Knockout	Mitochondrial inactivity, massive incremental accumulation of mitochondria	Temporary survival benefits but long-term sarcomeric damage in cardiomyocytes [90]
**YME1L**	Knockout	Distorted mitochondrial morphology	Heart failure [96]
**YME1L, OMA1**	Double Knockout	Normal mitochondrial morphology	Normal cardiac function [96]
**OMA1**	Mutation (mutation in OPA1’s OMA1 splice site)	Enlarged mitochondria, cristae disruption	Impaired cardiac systolic function [95]
**OPA1**	Mutation	Mitochondria larger, abnormal cristae	Myocardial hypertrophy [98]
		Mitochondria and cristae decrease	Late-onset cardiomyopathy [99]
**OPA1**	Overexpression	Normal mitochondrial morphology	Protect cardiomyocytes [100]

#### 3.1 Cardiac-related diseases

In heart failure patients, OPA1 expression decreased [91]. Right ventricular failure is marked by a reduction in the expression of genes associated with ETC and mitochondrial antioxidant, along with increased expression of genes that are markers of oxidative stress. In right ventricular failure mouse model, fusion protein OPA1 decreases, mitochondrial fission protein DRP1 trends to increase, and all ETC complexes activity reduced [92]. In a ventricular remodeling model triggered by surgical ligation of the coronary artery in Sprague-Dawley (SD) rats, protein analysis in cardiac homogenates, revealed that at 12 and 18 weeks after myocardial infarction, Fis1 expression increased by 80% and 31%, respectively. Conversely, MFN2 expression decreased by 17% and 22%. OPA1 expression remained unchanged at 12 weeks but decreased by 18% at 18 weeks, suggesting a delayed impairment in IM fusion [93].

OPA1, as a key mitochondrial dynamics regulatory protein, is crucial for heart development and function. OPA1 responds to the metabolic demands of the heart through its protein modifications, such as cleavage by OMA1 and YME1L [94]. In an OPA1 transgenic mouse model, the OMA1 splice site was deleted. Mice express normal OPA1 and mutant OPA1 that cannot be cleaved by OMA1. Mouse mitochondria showed greater heterogeneity, increased mean area and cristae disruption. The MMP of cardiomyocytes was significantly decreased, the oxygen consumption rate was decreased, and the cardiac systolic function was impaired, but it did not reach the criteria for heart failure [95]. In a mouse model characterized by specific deletion of YME1L in cardiomyocytes, abnormal splicing of OPA1 disrupts the balance of mitochondrial fusion and fission dynamics, thereby impairing the OXPHOS capacity of mitochondria and consequently reducing energy production. This energetic deficiency compromises the normal contractile and relaxation functions of cardiomyocytes, ultimately leading to cell death and heart failure. However, deletion of OMA1 in YME1L knockout cells prevents the abnormal processing of OPA1, thereby restoring normal mitochondrial morphology and cardiac function. These findings suggest that regulation of OPA1 splicing and mitochondrial dynamics may represent a promising therapeutic strategy for the treatment of heart failure [96]. In hearts with OXPHOS dysfunction, loss of OPA1 splicing worsens mitochondrial heart disease and affects the progression of cardiac hypertrophy [97]. Therefore, maintaining the normal processing of OPA1 is also crucial for heart health.

Research has been conducted using OPA1 mutant mouse models to analysis cardiac function. Two mouse models with mutations both resulting in the GTPase domain are lethal when homozygous, while heterozygous mutants can survive but exhibit certain disease phenotypes. One type of OPA1 heterozygous mutant mouse does not have a shortened protein, but the overall protein level is reduced by 50%. The heart function is not significantly different from that of normal mice under baseline conditions, but these mice have larger mitochondria with clusters of fused mitochondria and abnormal cristae structures. Additionally, the sensitivity of the mitochondrial permeability transition pore (PTP) opening to calcium ion accumulation is reduced. When subjected to pressure load through transverse aortic constriction (TAC), the hearts of OPA1 heterozygous mutant mice exhibit almost twice the hypertrophic response compared to wild-type mice [98]. The other mouse model, with a heterozygous mutation that produces a truncated form of the protein, results in a deficiency of antioxidant transcripts, increased ROS, and mitochondrial dysfunction. At 12 months, heart function shows significant abnormalities, including a decrease in left ventricular fractional shortening (LVFS), cardiac output, and myocardial cell contractility, leading to late-onset cardiomyopathy [99]. Overexpression of OPA1 can stimulate mitophagy, inhibit mitochondrial fission, and activate the MAPK/ERK signaling pathway, thereby enhancing the cell's antioxidant capacity and protecting cardiomyocytes from oxidative stress damage [100] (Table 1).

#### 3.2. Ischemia/reperfusion injury

Ischemia/reperfusion (I/R) injury, commonly seen in myocardial infarction, stroke, and peripheral vascular diseases, is formed by early ischemic damage followed by further damage caused by reperfusion. Ischemia leads to a decrease in intracellular ATP levels and cellular acidosis, while reperfusion triggers the massive production of ROS and infiltration of neutrophils, exacerbating tissue damage [101]. Studies have declared that in hearts with IRI, the amount of L-OPA1 decreases and OMA1 enzyme activity increases. Even after recovery of cardiac function with XJB-5-131 (XJB, a mitochondria-targeted antioxidant) and sanglifehrin A (SfA, a permeability transition pore inhibitor), L-OPA1 cleavage could not be prevented [102]. In a mouse model, cardiac-specific knockout of phosphoglycerate mutase 5 (PGAM5), a mitochondrial localized serine/threonine protein phosphatase, can reserve the decline of OPA1 in cardiomyocytes after IRI [103]. Changes in mitochondrial dynamics triggered by ischemia could be pivotal in limiting the therapeutic window for reperfusion and worsening the associated reperfusion injury [104].

Mice with moderate overexpression of OPA1 developed a non-pathological cardiac hypertrophy in 9 months and showed protective effects against IRI. Additionally, these mice were protected from denervation-induced muscle atrophy and Fas-induced hepatocyte apoptosis. The protective effects of OPA1 overexpression in various tissue injury models are attributed to its ability to stabilize the mitochondrial cristae structure, thereby reducing the outflow of Cyt c and the production of ROS [105]. Silent mating type information regulation 2 homolog-3 (SIRT3), a deacetylase primarily found in the mitochondrial matrix, affects the function of OPA1 through deacetylation and has a significant impact on maintaining mitochondrial function and regulating the balance of mitochondrial dynamics. SIRT3 targets the i-AAA protease YME1L1, regulates the cleavage of the mitochondrial protein OPA1, increases the level of L-OPA1, stimulates mitochondrial fusion, consequently reducing oxidative stress and ER stress, and enhances the kidney's resistance to IRI [106]. The mitochondrial calcium uniporter (MCU) is upregulated in expression during I/R, promoting the expression and activation of calpain-1/2, the main subtypes of calpain expressed in cardiomyocytes. These proteins inhibit mitochondrial fusion and autophagy by reducing the expression of OPA1, causing mitochondrial swelling and disorganization of cristae morphology, decreasing ATP production, and leading to myocardial injury and apoptosis. Inhibition of MCU function can reduce the area of myocardial infarction and cardiomyocyte apoptosis, and restore mitochondrial fusion and autophagy [107]. Therefore, OPA1 is a cornerstone of regulating mitochondrial dynamics and health, setting it as a notable priority for averting and treating IRI like myocardial infarction.

### 4. The impact of OPA1 on neurogenesis, neurodegeneration, and neurological Diseases

The term “adult neurogenesis” refers to the continuous production of functional neurons in certain adult brain zones, which can join preexisting neural networks and play key roles in learning and memory. Cognitive decline is linked to impaired neurogenesis [108]. Neural stem cells (NSCs) act as the wellspring of new neurons. Quiescence is the reversible phase of cell cycle arrest, where cells have low metabolic activity, are highly sensitive to the local signaling environment, and potential to be triggered by various physiological stimuli. In conditions of injury or disease, the quiescent state of NSCs may be activated to meet the brain's repair needs. The balance of activity and quiescence of NSCs in the adult brain determines both the speed of neuron creation and the ongoing preservation of stem cells, as well as how the brain generates neurons as it ages. The number of NSCs and their capacity for neurogenesis are age-related deterioration, and they react to activation signals more slowly [109]. Mitochondrial dysfunction, including insufficient energy supply, imbalance of mitochondrial dynamics, autophagy disorders, and inflammation, are observed in a variety of neurodegenerative diseases [110–112]. Therefore, ensuring mitochondrial health and functionality is essential in preventing and managing these conditions.

#### 4.1. OPA1 in neurogenesis and maintenance of NSCs

The expression of OPA1 is upregulated on the course of neural differentiation. OPA1 regulates the fate of NSCs by affecting mitochondrial dynamics. The absence of OPA1 leads to impaired neurogenesis in the adult hippocampal dentate gyrus. This impairment exhibits traits of decreasing in the activation and proliferation capacity of NSCs, resulting in a reduced number of newly generated neurons [113]. The absence of OPA1 also affects the proliferation capacity of type II neuroblasts (NBs) and mature intermediate precursor cells (INPs), causing a drop in mature INPs, ganglion mother cells (GMCs), and neurons. The loss of OPA1 alters mitochondrial morphology and function, provoking reduced Notch signaling in type II NB lineage. This process can be inhibited by a mutant form of Drp1, indicating mitochondrial fusion can partially restore the differentiation defect [114]. The activity and functional state of OPA1 can affect the fate of NSCs, including whether they remain undifferentiated, differentiate into specific types of neurons or supporting cells, or participate in tissue repair and regeneration processes. Acute knockdown of OPA1 in NSCs leads to mitochondrial fragmentation, a change that impairs the self-renewal capacity of NSCs, resulting in a decrease in cell numbers with age. Furthermore, OPA1 plays a crucial role in regulating mitochondrial dynamics and ROS levels. This regulation subsequently activates the nuclear factor erythroid 2-related factor 2 (NRF2), a key transcription factor in the cellular antioxidant response. Upon activation, NRF2 translocates to the nucleus and induces the expression of antioxidant genes. This activation cascade results in a series of gene expression changes associated with the self-renewal and differentiation of NSCs, ultimately influencing the nuclear transcriptional program of these cells [115]. Haploinsufficiency of OPA1 leads to abnormal DNA methylation patterns in NSCs, in particular, reduced expression of OPA1 gives rise to the dysfunction of crucial transcription factors in NSCs, such as FoxG1, which is vital for the differentiation of neural lineages. Haploinsufficiency of OPA1 induces an oxidative stress in NSCs, characterized by increased levels of ROS, which may also be related to its methylation regulation [116]. In the absence of OPA1, mitochondrial dysfunction activates the integrated stress response (ISR) pathway, particularly the expression of activating transcription factor 4 (ATF4), which promotes the survival of NSCs when confronted with metabolic stress challenges. Knockout of ATF4 accelerates cell death in OPA1-deficient NSCs, while ATF4 overexpression promotes proliferation and survival. It has also been found that ATF4 acts as vital for NSCs survival and function under stress conditions through its target gene solute carrier family 7 member 11 (Slc7a11) mediated glutathione production [113].

#### 4.2. OPA1 and age associated with nerve damage

Presbycusis, or age-related hearing loss (ARHL), refers to a progressive bilateral sensorineural hearing decline that occurs as part of the aging process. Recently research found that disorders in the quantity and acetylation activity of OPA1 are key factors in the onset and development of ARHL. Activating the SIRT3/OPA1 signaling pathway can ameliorate the imbalance of mitochondrial dynamics in cochlear hair cells of aged mice, slow down hair cells aging, and reduce hearing loss [117]. Age-related macular degeneration (AMD) is a common eye condition that brings about blindness, which is becoming more prevalent as the population ages. Inducing retinal damage in mice through blue light exposure creates a model of AMD. The model shows that mitochondrial fusion is impaired [118, 119].

#### 4.3. OPA1 and stroke

Ischemic stroke accounts for a significant number of disabilities and fatalities among adults globally. For those with ischemic stroke, reperfusion therapy is the accepted clinical protocol. Although vital for the preservation of ischemic tissue, reperfusion may also lead to increased neuronal harm as a result of a chain of molecular events. Mitochondrial dysfunction is major in mediating the pathological processes of IRI. OPA1 dysfunction may cause the loss of MMP, increase mitochondrial ROS production, release apoptotic factors like Cyt c, and activate apoptotic pathways [120]. In P. R. China, according to the latest survey reports, the population of individuals aged 40 and above suffering from stroke has reached tens of millions, with ischemic stroke accounting for nearly 90%, and the population at risk of developing one is getting younger. Currently, endovascular thrombectomy and tissue plasminogen activator (tPA) are accepted treatment for ischemic stroke, whereas the window time of their treatment limits their use. Mitochondrial dysfunction is a hallmark feature in I/R-induced neuronal death, involving failure in energy production, oxidative stress, and calcium ion homeostasis imbalance, and it is evident in both ischemic and hemorrhagic stroke scenarios. Mitochondrial dynamics is vital for preserving mitochondrial integrity and function, it holds a significant role in the progression of ischemic stroke. MFN1/2 and OPA1 are key regulatory proteins for mitochondrial fusion, maintaining the integrity and function of the mitochondrial network. The expression of these proteins decreases after stroke, leading to mitochondrial dysfunction and neuronal death [121, 122]. Studies have revealed that neuronal mitochondrial fission triggered by ischemia is coupled with the cleavage of OPA1 at the S1 site by OMA1, which exacerbates neuronal mitochondrial fragmentation and damage in a GTPase-dependent process and contributes to IRI. In the middle cerebral artery occlusion/reperfusion (MCAO/R) mouse model, overexpression of S1-OPA1 exacerbates mitochondrial damage [123]. Overexpressing OPA1 in a transgenic manner can "tighten" the CJs, curb the discharge of Cyt c, and shield the brain against injury caused by ischemia [105]. The upregulation of SIRT3 can increase the expression of OPA1 after cerebral IRI, and then reduce the volume of cerebral infarction [124]. Changes in OPA1 expression are closely associated with the modulation of mitochondrial morphology and function, thus playing a crucial part in stroke onset and protection.

#### 4.4. OPA1 and neurodegenerative diseases

AD (Alzheimer’s disease) stands out as a highly common form of neurodegenerative illness. The diagnosis of AD is defined by deposition of amyloid beta (Aβ) and phosphorylated tau protein [125]. Studies have elucidated lower levels of OPA1 in AD mouse models, but boosting OPA1 expression can lessen the mitochondrial damage and neuronal apoptosis caused by Aβ1-42 [126, 127]. However, the cleavage of OPA1 does not alter the phosphorylation of tau, and the phosphorylation of tau does not induce or correlate with the hydrolysis of OPA1. The post-translational modification of tau protein and the adjustment of the OMA1-OPA1 pathway are two independent processes in AD [128]. By blocking mitochondrial complex Ⅰ, neurotoxins associated with Parkinson's disease (PD) cause the increase of ROS and disassembly of OPA1 oligomeric complexes, which normally maintain the integrity of mitochondrial CJs. The disassembly leads to significant structural abnormalities of mitochondria, including cristae disorganization and swelling, loss of matrix density. Overexpression of OPA1 can alleviate the mitochondrial structural remodeling associated with complex I inhibition, safeguard the mitochondrial membrane’s integrity, and curb the discharge of Cyt c, thus preventing the deterioration of dopaminergic neurons attributed to complex Ⅰ deficiency [129]. In the Huntington's disease (HD) mouse model, mitochondrial cristae disruption, and the decreased transcription levels of OPA1 in the cortex and striatum, along with a reduced ratio of long isoforms to short isoforms, affecting OPA1 oligomerization [130]. Overexpressing of OPA1 or using inhibition of DRP1 improved mitochondrial morphology and decreased apoptotic sensitivity in HD cell models [131]. Prion diseases are defined by the conversion of cellular prion protein PrP^C^ into protease-resistant misfolded form PrP^SC^ and have a relationship with a variety of mitochondrial damages. Downregulation of OPA1 expression is observed in prion disease models, while overexpression of OPA1 can alleviate the fragmentation and remodeling of cristae structure, mitochondrial dysfunction, mtDNA depletion, and neuronal cell apoptosis [132]. Diseases related to OPA1 affect multiple systems in the body, including the central nervous system, the peripheral and autonomic nervous systems, demonstrating its key role in neurobiology [133].

#### 4.5. Neurological diseases associated with OPA1 mutations

A single mutated allele of the OPA1 gene is sufficient to set off the disease phenotype. Mutations in the OPA1 gene have been identified as associated with optic atrophy on chromosome 3q28. This autosomal dominant optic atrophy (ADOA) is a disease that leads to damage to the optic nerve and a decline in vision [134–136]. Researchers have mimicked the human pathological mutation in a heterozygous OPA1 mutant mouse model carrying a termination codon at position 285 at the start of the GTPase domain. Reduced OPA1 protein levels in these mice cause selective loss of subunits of complex IV of the respiratory chain. Partial deficiency of OPA1 results in significant resistance to apoptosis induced by ER stress, but sensitivity to other apoptotic stimuli is either normal or increased. The findings rule out the possibility of harmful dominant-negative or gain-of-function effects associated with OPA1 mutation, confirming that haploinsufficiency of OPA1 is the mechanism of mitochondrial dysfunction in ADOA [137].

Mutations in the OPA1 gene not only lead to ADOA but can also develop into a more severe condition referred to as ADOA plus syndrome. This is accompanied by sensory neurosensory hearing loss, ataxia, axonal sensory-motor polyneuropathy, chronic progressive external ophthalmoplegia, and mitochondrial myopathy. A multitude of mtDNA deletions have been found in all skeletal muscles of these patients, suggesting that specific missense mutations in the OPA1 gene are related to the upkeep of mtDNA integrity [138]. Fibroblasts from patients exhibit increased mitochondrial fragmentation and autophagy [139]. The OPA1^delTTAG^ mutant mouse model is utilized to replicate ADOA plus syndrome and investigate auditory neuropathy spectrum disorder (ANSD), the mutant mice exhibit adult-onset progressive auditory neuropathy. Ultrastructural analysis reveals that the mutant mice selectively lose sensory inner hair cells and experience progressive degeneration of the axons and myelin of spiral ganglion neurons [140].

**Figure 5.**
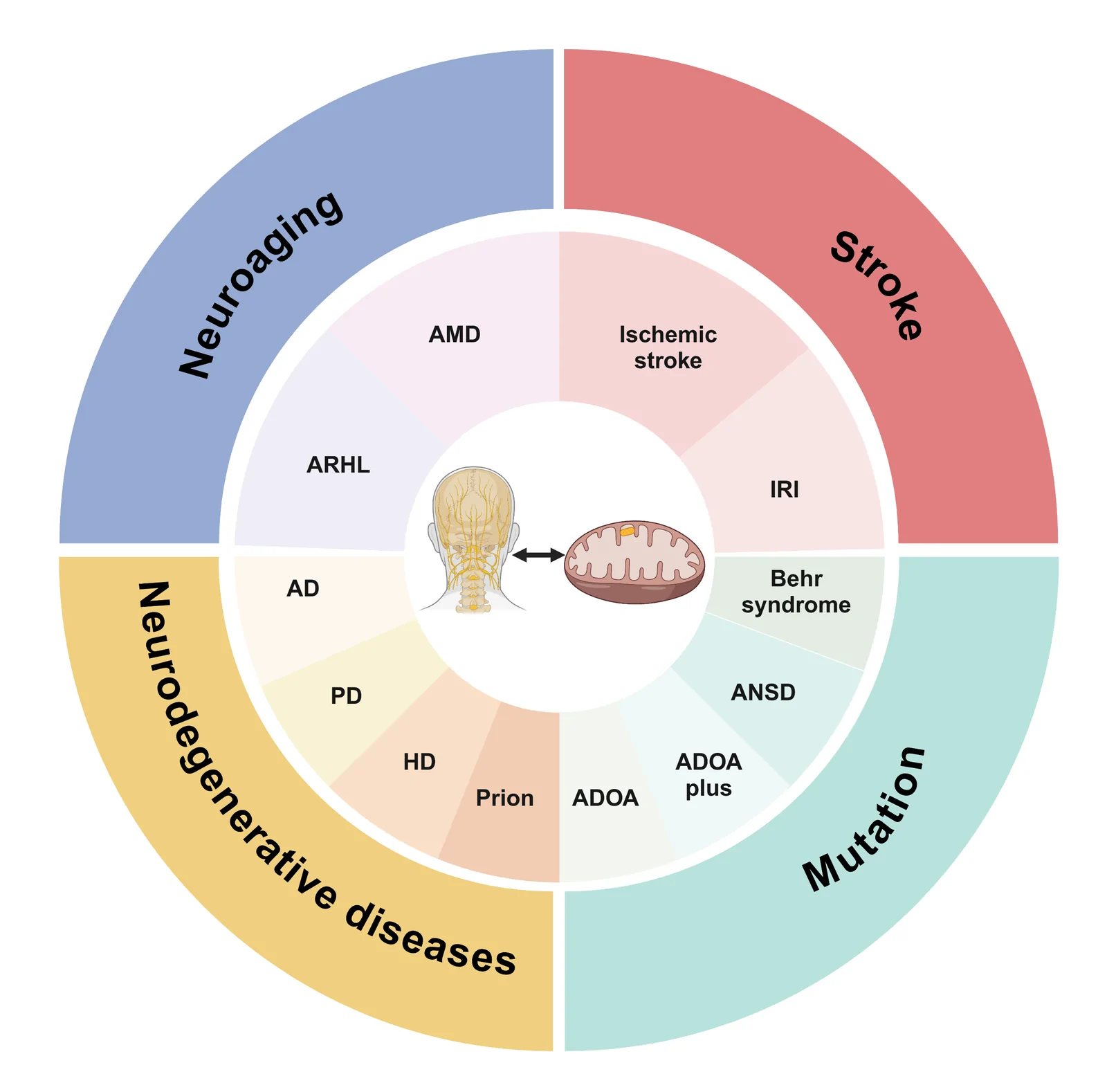
**Neurological diseases caused by OPA1 abnormalities.** Abnormalities in OPA1 expression of function have been implicated in several neurological diseases, as illustrated by the figure, which highlight some of the key conditions associated with OPA1 dysfunction.

Mutations in OPA1 are predominantly localized in the GTPase domain, and the risk of developing multisystem neurological diseases directly increased when mutations occur within this domain [141]. A case report describes a young man exhibiting progressive upper limb dystonia, spastic quadriplegia, and ataxia. The whole exome sequencing (WES) identified a novel variant c.2357A>G in the OPA1 gene, located outside the GTPase domain of the OPA1 protein's dynamism structure domain. Distinct from mutations occurring in the GTPase domain of OPA1, this new mutation did not lead to severe OXPHOS impairment or mtDNA depletion, but is was associated with basal ganglia atrophy and reduced glucose metabolism, manifesting as multiple movement disorders [142]. Dual heterozygous alterations in the OPA1 gene can cause Behr syndrome, which is characterized by severe vision impairment, cerebellar ataxia, peripheral neuropathy, digestive issues, deafness, as well as spastic paraparesis [143, 144]. Mutations in the OPA1 gene manifest a variety of neurological diseases (Fig. 5). Thus, studying OPA1 gene mutations aids in the early diagnosis and treatment of these disorders.

### 5. OPA1 and tumor dynamics

According to the latest global cancer statistics, roughly 20 million individuals were newly diagnosed with cancer, while nearly 9.7 million died from cancer in 2022. Cancer is not only a stumbling block to the improvement of population life expectancy, but it also has a significant economic impact [145]. As age increases, so does the risk for cancer development, and as one gets older, the function of the immune system gradually declines, resulting in an increased incidence of cancer [146]. The occurrence and development of tumors is a sophisticated process, encompassing both the proliferation of tumor cells themselves and the tumor microenvironment (TME). Cells in the TME, such as tumor-associated macrophages (TAMs), myeloid-derived suppressor cells (MDSCs), regulatory T cells (Tregs), and B cells, as well as the cytokines are the main mediators of intercellular communication in the TME. They promote the development of cancer by stimulating different carcinogenic signaling and enhancing immune evasion [147]. Tumor cells tend to produce energy through glycolysis, even under oxygen-rich conditions, this phenomenon is known as the Warburg effect. This impact promotes an increase in intracellular alkaline pH, which is a driving force for cancer cell proliferation and invasiveness [148]. The function of mitochondria in cancer treatment are complex, involving energy metabolism, cell death regulation, ROS production, autophagy processes, drug effects [149]. Mitochondria, ROS activation serves as a mechanism to trigger apoptosis, laying the strong foundation for cancer treatment. Drugs targeting mitochondrial bioenergetic pathways can induce the death of cancer cells, opening up new therapy options [150].

#### 5.1. OPA1 and tumor cells

OPA1 shows high levels of expression or undergoes mutations in numerous tumor cells, and there is a strong correlation between its expression levels and patient survival rates, as well as immune cell infiltration. In some types of tumors, a notably poorer overall survival rate is associated with patients who have high OPA1 expression levels compared to those with low levels, and OPA1 mutations are significantly associated with improved patient prognosis [151]. By using hepatocellular carcinoma (HCC) cell lines and cholangiocarcinoma (CCA) tumor organoids cultured *in vitro*, it was discovered that knocking down OPA1 and MFN1 genes with a lentiviral vector, can inhibit the mitochondrial fusion process, leading to the suppression of cellular proliferation *in vitro* and tumorigenesis *in vivo*. Whole-genome transcriptome analysis showed that inhibiting fusion mainly impacted cellular metabolic pathways. Blocking mitochondrial fusion decreased the oxygen uptake and ATP synthesis in cancer cells [152]. OPA1 deficiency disrupts the morphology of the cristae, inhibits the assembly and activity of the ETC, and thus inhibits the proliferation of tumor cells by interfering with Nicotinamide Adenine Dinucleotide (NAD^+^) regeneration. In kirsten ratsarcoma viral oncogene homolog (K-Ras) mutated lung adenocarcinoma, the absence of OPA1 inhibits tumor growth, whereas the absence of DRP1 does not affect the growth of K-Ras driven lung adenocarcinoma *in vitro* and *in vivo*, but it can rescue the decline in ETC function and reduced cell proliferation caused by the absence of OPA1 [153]. Overexpression of OPA1 enhances the growth and migration of lung adenocarcinoma. The amplification of OPA1 and MFN1 copy numbers occurs together in lung cancer cell tissues, and high levels of OPA1 are connected to a worsened prognosis. The absence of OPA1 leads to a decrease in MMP and an increase in the release of Cyt c, thereby causing cancer cell apoptosis. Mechanistically, damaged mitochondria activate the apoptotic signaling pathway, causing cells to stagnate in the G1 phase and accelerating apoptosis [154]. N6-methyladenosine (m6A) promotes mitochondrial fusion by inducing GSH synthesis and OPA1 expression, promoting the proliferation of cancer cells and the progression of colorectal cancer (CRC) [155]. The above evidence proves that mitochondrial dynamics exert a significant influence on the regulation of cancer cell growth and invasiveness.

#### 5.2. OPA1 and angiogenesis

In tumor angiogenesis, OPA1 in endothelial cells is crucial for blood vessel formation, tumor growth, and metastasis. OPA1 regulates tumor angiogenesis during angiogenic stimulation by modulating calcium ion levels and the NF-κB signaling pathway. In another perspective, as a principal protein in IM fusion, OPA1 activation aids in the maintenance of the morphology and function of mitochondria. Changes in mitochondrial morphology may affect the cell's metabolic state and energy supply, thereby influencing angiogenesis. The absence of OPA1 can promote the normalization of tumor blood vessels, as seen by reduced vessel diameter and increased pericytes covering, potentially reduce tumor vascular leakage and the escape of tumor cells [156]. OPA1 thus plays an important role in tumor angiogenesis.

#### 5.3. OPA1 and tumor immunity

The role of OPA1 in tumor immunity is multifaceted. On the one hand, OPA1 can assist in anti-tumoral responses by modulating with immune cell function and activity. On the other hand, under certain circumstances, OPA1 may contribute to tumor cell survival by enabling immune evasion and promoting tumor progression.

Mitochondria are indispensable in regulating the function and survival of T lymphocytes. CD137 is a co-stimulatory receptor in the tumor necrosis factor receptor (TNFR) family, whose function can be used for cancer immunotherapy. CD137 co-stimulation activates the mTOR signaling pathway and increases the mitochondria quality in T cells in an OPA1-dependent manner, characterized by an increase in mitochondrial volume and enhanced MMP, leading to improved respiratory capacity of T cells. When OPA1 is silent, the T cell response to CD137 co-stimulation is impaired. Furthermore, the expression of OPA1 is essential for T cells to produce effector molecules such as interferon-gamma (IFN-γ) and tumor necrosis factor-alpha (TNF-α), which are critical for T cell-mediated anti-tumor responses. By promoting mitochondrial fusion and enhancing mitochondrial function, OPA1 has a significant impact on the anti-tumor effects of T cells and is a key molecule linking CD137 co-stimulation to the metabolic reprogramming of T cells [157]. The role of OPA1 in T cell tumor immunity is also regulated by the sentrin-specific protease 1- sirtuin 3 (SENP1-SIRT3) signaling pathway. SENP1 initiates the deacetylase activity of SIRT3 within T cell mitochondria, causing a decrease in the acetylation of mitochondrial metalloprotease YME1L1. The deacetylation of YME1L1 inhibits its proteolytic activity toward OPA1, thereby preserving mitochondrial fusion and enhancing the metabolic capacity of T cells. Specifically, this process promotes OXPHOS and FAO, which collectively provide an increased energy supply for T cells. As a result, T cell survival, memory T cell formation, and anti-tumor immune responses are significantly enhanced [158].

OPA1 additionally contributes to regulating the function of invariant natural killer T (iNKT) cells. INKT cells with OPA1 deficiency exhibit a shift in cellular subpopulations, with a reduced frequency of S3 cells and increased frequencies of S1 and S2 cells. These subpopulations represent distinct stages of iNKT cell development, with S1 marking the earliest stage and S3 representing the most mature stage. Notably, in OPA1-deficient cells, there are significant changes in mitochondrial mass, MMP, and mitochondrial volume in S3 iNKT cells, the proportion of Ki67^+^ proliferating cells is increased. These findings suggest that OPA1 enhances the quiescence of iNKT cells, particularly at the S3 stage, during development [159]. Quiescent iNKT cells have great potential in tumor immune regulation.

When key factors such as metastasis-associated protein 2 (MTA2), methyl-CpG binding domain protein 2 (MBD2), chromodomain-helicase-DNA-binding protein 4 (CHD4), or B-cell lymphoma/leukemia 11B (BCL11B) are knocked out in T cells, these cells upregulate NK cell-associated receptors and transcription factors, thereby transitioning into cell type resembling NK cells, called induced T-to-NK cells (ITNKs). OPA1-mediated mitochondrial fusion, along with the enhancement of OXPHOS activity, increases the cellular acetyl-CoA levels. This elevation of acetyl-CoA subsequently promotes the reprogramming efficiency and anti-tumor effects of ITNKs by regulating the H3K27 acetylation of specific genomics targets [160].

T cell immunoglobulin and mucin domain molecule 4 (TIM-4) regulates L-OPA1 protein through the PI3K/AKT signaling pathway, promoting mitochondrial fusion. TIM-4 interacts with annexin A2 (ANXA2) to promote the activation of the PI3K/AKT signaling pathway, enhancing OXPHOS in lung cancer cells and accelerating tumor progression [161]. OPA1, tumor cells also influence their communication with immune cells, having an influence on the tumor initiation and development. The absence of OPA1 impairs mitochondrial dynamics and blocks respiratory function, increasing the sensitivity of non-small cell lung cancer (NSCLC) tumor epithelium to CD8^+^ T cells. Sequencing data reveals that OPA1^-/-^ tumor epithelial cells show significantly enhanced communication with CD8^+^ T cells. OPA1 supports mitochondrial metabolism, ensuring sufficient ATP production to support their rapid proliferation, and it may also regulate the activity of immune cells by affecting metabolic by products, thereby affecting the tumor microenvironment. The absence of OPA1 leads to mitochondrial dysfunction, cell cycle arrest, and subsequently affects the antitumor immune response. This points to the possibility that tumor cells with OPA1 positive might evade immune evasion [154].

In most age-related diseases, OPA1 is recognized for its beneficial effects, and its deficiency leads to abnormal mitochondrial morphology and dysfunction, thereby contributing to corresponding disease phenotypes. However, in the context of tumors, it acts as a double-edged sword. Within immune cells, it sustains mitochondrial integrity to support anti-tumor immunity. Conversely, in malignancies such as carcinomas and solid tumors, OPA1 overexpression is linked to poor prognosis [162]. Tumor cells exploit OPA1 to promote several oncogenic processes. For instance, OPA1 facilitates angiogenesis through hypoxia-responsive pathways, sustaining metabolic reprogramming essential for survival by balancing oxidative phosphorylation and glycolysis. Additionally, OPA1 enhances cell proliferation via mitochondrial biosynthetic pathways, and evades immune surveillance by impairing host immune recognition and cytokine-mediated clearance. This dichotomy stems from OPA1's fundamental role in maintaining mitochondrial homeostasis, a process co-opted by tumors through metabolic plasticity and microenvironmental adaptation. By enhancing mitochondrial resilience, OPA1 fosters tumor stemness, proliferative vigor, and stress tolerance, thereby paradoxically driving malignancy progression and therapeutic resistance despite its protective role in degenerative contexts.

### 6. Therapeutic strategy for OPA1-related disease intervention

OPA1 plays a critical role in numerous tissues and organs, and aberrant expression is closely associated with a variety of diseases. Therefore, research and treatment focused on OPA1 also become increasingly important.

#### 6.1. Gene therapy

ADOA is primarily a hereditary disease rather than a typical age-related disease. Typically, it arises from mutations in the OPA1 gene, resulting in the gradual degeneration of optic nerve fibers and subsequent vision loss. Since mitochondrial dysfunction also has a major impact on many age-related diseases, exploring gene therapy methods for OPA1 may help in understanding and treating OPA1-related age-associated diseases. In case of ADOA resulting from the absence or mutation of OPA1, the phenotype can be ameliorated by supplementing or delivering functional OPA1. For instance, intravitreal injection of adeno-associated virus (AAV) containing human OPA1 cDNA, governed by the cytomegalovirus promoter has been demonstrated to diminish the severity of retinal ganglion cell degeneration associated with OPA1 [163]. Furthermore, gene correction technologies such as clustered regularly interspaced short palindromic repeats (CRISPR)/CRISPR associated protein 9 (CRISPR/Cas9) have verified great potential, two guide RNAs (gRNAs) targeting the OPA1 c.1334G>A mutation, along with a single-stranded DNA (ssDNA) repair template were designed for gene correction. The corrected induced pluripotent stem cells (iPSCs) showed recovery of mitochondrial homeostasis at the morphological, functional, and genetic levels, including restored wild-type (WT) mtDNA levels and alleviated sensitivity to apoptotic stimuli [164]. Constructing cell models using fibroblasts obtained from patients and healthy controls via skin biopsies, engineered U1 small nuclear ribonucleoproteins (U1 snRNAs) targeting specific regions of the OPA1 gene were shown to significantly reduce exon 10 skipping and enhance the expression levels of normal transcripts [165]. In addition, Maloney et al. constructed expression vectors for OPA1 isoforms 1 and 7, assessing their therapeutic effects in a cellular model of mitochondrial dysfunction. Both isoforms were capable of ameliorating the degeneration of retinal ganglion cells caused by OPA1 gene knockout and mitochondrial dysfunction, thereby preserving spatial visual function. It is noteworthy to rigorously regulate the levels of OPA1 expression to optimize therapeutic benefit [166].

OPA1-targeted therapies may vary significantly depending on the specific disease or patient phenotype. For instance, OPA1 defects cause mitochondrial myopathy, gene delivery using AAV serotypes with muscle-specific tropism could be prioritized [167]. Pre-existing immune responses against wild-type AAV can compromise transduction efficiency, as neutralizing antibodies may block vector entry into target cells, particularly in patients with prior AAV exposure [168]. However, neutralizing antibodies can pose risks to patients by reducing transgene expression and therapeutic efficacy. To address this, corticosteroids are commonly used in clinical trials to prevent or mitigate immune responses. Patients can be screened for neutralizing antibodies and treated with plasmapheresis before AAV administration. Emerging approaches, such as using endopeptidases to degrade IgG and lower neutralizing antibody levels, are also being explored [169–171]. A major challenge in gene therapy is the timing of treatment. Although AAV vector-mediated gene replacement therapy shows potential in preventing disease development, its benefits are limited for patients who already exhibit symptoms. However, more excitingly, several studies have demonstrated that AAV gene replacement therapy can ameliorate or slow the established phenotypes of mitochondrial diseases [172, 173].

CRISPR-Cas9 uses designed sgRNA to direct the Cas9 protein to target sites for gene correction via Homology-Directed Repair (HDR) or base editing. Challenges include off-target effects, where Cas9 may cleave unintended DNA, and difficulties in delivering Cas9 and sgRNA efficiently. Additionally, the Cas9 protein can provoke immune responses, affecting editing efficiency and safety [174]. To address these challenges, personalized approaches are essential. This includes selecting AAV serotypes based on patient immune profiles, refining CRISPR designs to minimize off-target risks, and optimizing delivery systems for specific tissues [175]. AAV vector-based CRISPR therapies are highly attractive due to their ability to enable gene therapy for genes larger than the AAV packaging capacity, without the need to split the gene and deliver fragments in multiple AAV particles. By overcoming these hurdles, these technologies hold promise for developing effective and safe treatments for OPA1-related mitochondrial disorders.

#### 6.2. Pharmacological therapy

In addition to gene therapy, some drugs have been confirmed that can restore mitochondrial morphology and function by influencing OPA1 expression. Pectolinarigenin (PLG), a natural flavonoid, improves mitochondrial function and reduces cell apoptosis by activating the SIRT3/OPA1 axis, which regulates mitochondrial fusion. It shows potential therapeutic effects on the pathological processes related to amyotrophic lateral sclerosis (ALS) and frontotemporal dementia C9-ALS [176]. Doxorubicin (DOX), a major tetracycline antibiotic, alleviates ROS-induced mitochondrial fission and depolarization in H9c2 cardiomyocytes. In the model of heart failure caused by isoproterenol (ISO), it reduces the gravity of heart failure by modulating the expression of key regulators of mitochondrial fusion and fission, such as MFN2, OPA1, and DRP1 [177]. Melatonin regulates the expression of OPA1 by activating the Hippo/yes-associated protein (Yap) signaling pathway. Stimulating the expression of OPA1 promotes mitochondrial fusion to counteract mitochondrial fission in cardiac IRI. This maintains normal mitochondria morphology and function, ensuring effective energy metabolism and ATP production, which is crucial for the cardiomyocytes survival during ischemia-reperfusion conditions [178]. PT320, a sustained-release formulation of the GLP-1 receptor agonist Exenatide, offers protection in the MitoPark (MP) mouse model of progressive PD. PT320 reduces the expression of mitochondrial fission protein 1 (FIS1) and increases OPA1 expression, contributing to mitochondrial homeostasis and reducing Cyt c release. Early governance of PT320 may serve as a neurological preservation therapy for PD [179]. Sodium glucose cotransporter type 2 (SGLT2) inhibitor Empagliflozin upregulates OPA1 expression, preventing renal IRI by inhibiting mitochondrial fission and reducing inflammation [180]. Meriolin derivatives, a novel category of kinase inhibitors, exhibit significant cytotoxic potential by promoting the cleavage of OPA1, thereby influencing mitochondrial dynamics and apoptosis. These compounds exhibit potent apoptotic capacity in Jurkat leukemia and Ramos lymphoma cells [181].

Additionally, traditional Chinese medicines have shown potential in regulating OPA1 expression and mitochondrial function. Paeonol, derived from the Paeonia suffruticosa, upregulates the expression of OPA1 through activating the CK2α-Stat3 signaling pathway, thereby stimulating mitochondrial fusion, inhibiting mitochondrial oxidative stress, and maintaining mitochondrial respiration and cardiac function in diabetic hearts and high-glucose-treated cardiomyocytes [182]. Celastrol, an active triterpenoid compound extracted from Thunder God Vine, demonstrate significant anti-tumor angiogenesis effects. Celastrol suppresses the phosphorylation of STAT3, leading to a decrease in OPA1 expression, bringing about mitochondrial fragmentation and NF-κB activation, ultimately inhibiting tumor angiogenesis *in vitro* and *in vivo* [183].

Gene therapy and pharmacological therapy represent complementary approaches in treating OPA1-related mitochondrial disorders. Gene therapies directly eliminate genetic aberrations, restoring normal protein function and mitochondrial architecture. Simultaneously, pharmacological interventions enhance mitochondrial function by reestablishing mitochondrial network, activating metabolic pathways, reducing oxidative stress [184]. Given the critical role of OPA1 in mitochondrial dynamics, understanding how these therapeutic approaches can be integrated is essential. OPA1 abnormalities impair mitochondrial fusion and fission, leading to mitochondrial dysfunction and consequently triggering mitochondrial related diseases. While correcting OPA1 expression is essential for addressing these issues, it should be noted that pharmacological interventions targeting specific mitochondrial disorders can synergistically facilitate functional recovery. For instance, when OPA1 defects cause mitochondrial myopathy, considering combination therapy incorporating gene correction along with niacin administration could enhance muscular performance [185]. Similarly, in cases where OPA1 dysfunction manifests mitochondrial myopathy, encephalopathy, lactic acidosis, and stroke (MELAS)-like symptoms, concurrent use of L-arginine or taurine supplementation could be considered during gene therapy may reduce stroke incidence and severity [186, 187]. By improving metabolic efficiency and reducing free radical damage, the combined use of pharmacological approaches may aid in cellular repair and regeneration. In the future, we should continue to delve deeper into the pathophysiological processes underlying the various tissue changes and clinical symptoms caused by mitochondrial disorders, and create advanced diagnostic and therapeutic approaches, accelerate their transition into clinical practice.

## Conclusions and Future Perspectives

OPA1 is a crucial mitochondrial fusion protein that plays a vital role in maintaining mitochondrial morphology and function. Disruption of OPA1 leads to impaired mitochondrial fusion, abnormal cristae structure, and subsequent alterations in MMP and oxidative capacity. It also regulates the release of Cyt c and calcium ions, increasing cellular sensitivity to apoptotic signals, ultimately resulting in cell damage, apoptosis and necrosis [188–190]. Specifically, deficiency or mutations in OPA1 can cause mtDNA depletion, leading to defects in the assembly or dysfunction of mitochondrial respiratory complexes. In addition to protecting cells from apoptosis or other forms of cell death, the latest research reveals that OPA1 promotes ferroptosis in addition to its protective effect on cells. In OPA1-deficient cells, the upregulation of the xCT-GSH-GPx4 antioxidant axis combined with activation of the ISR pathway significantly diminishes lipid peroxidation levels. This attenuation of oxidative lipid damage reduces key ferroptosis drivers, thereby conferring cellular resistance to ferroptosis [191]. The pleiotropic effects of OPA1 appear context-dependent, with its functional dichotomy being governed by specific cellular microenvironments and stress signaling dynamics. While OPA1 predominantly serves as a mitochondrial protector under physiological conditions, particular pathological states or distinct cell lineages may experience OPA1-mediated potentiation of ferroptosis pathways, ultimately manifesting detrimental outcomes. This paradoxical behavior underscores the intricate regulatory network through which mitochondrial dynamics proteins modulate cell fate decisions under stress conditions.

Considering the important role of OPA1 in disease, further investigation into the role of OPA1 in these diseases may help to uncover its potential as a therapeutic target and biomarker. While gene therapy and pharmacological therapy show promise in preclinical research, they remain in the nascent stages of clinical trials. To enhance the translational potential of pharmacological findings, future directions should prioritize the development of more pathologically relevant human disease models and optimization of clinical trial designs. Investigating the pathological changes and stress responses in tissues and cells caused by mitochondria disorders can help elucidate the genotype-phenotype correlations and tissue-specific mechanisms of these diseases. These efforts are essential to accelerate the translation of basic research findings into clinical applications.
